# A multilocus assessment reveals two new synonymies for East Asian *Cyclommatus* stag beetles (Coleoptera, Lucanidae)

**DOI:** 10.3897/zookeys.1021.58832

**Published:** 2021-03-02

**Authors:** Jiao Jiao Yuan, Dan Chen, Xia Wan

**Affiliations:** 1 Department of Ecology, School of Resources and Engineering, Anhui University, 111 Jiulong Rd., Hefei 230601, China Anhui University Hefei China; 2 Anhui Province Key Laboratory of Wetland Ecosystem Protection and Restoration, Hefei 230601, China Anhui Province Key Laboratory of Wetland Ecosystem Protection and Restoration Hefei China

**Keywords:** East Asia, Lucanidae, molecular phylogeny, morphology, new synonym, species delimitation

## Abstract

*Cyclommatus scutellaris* Möllenkamp, 1912, *Cyclommatus elsae* Kriesche, 1921 and *Cyclommatus tamdaoensis* Fujita, 2010 are East Asian stag beetle species with long-debated taxonomic relationships due to high intraspecific morphological variability. In this study, we applied multilocus phylogenetic analyses to reassess their relationships. Two mitochondrial genes (16S rDNA, COI) and two nuclear genes (28S rDNA, Wingless) were used to reconstruct the phylogeny through the Bayesian inference (BI) and Maximum Likelihood (ML) methods. Both topologies supported two clades: the clade *C. scutellaris* was sister to the clade (*C. elsae* + *C. tamdaoensis*) with the subclade *C. tamdaoensis* embedded in the subclade *C. elsae*. The Kimura 2-parameter (K2P) genetic distance analysis yielded a low mean value (≤0.035) among the three taxa, which was well below the minimum mean value between other *Cyclommatus* species (≥0.122). We also compared the accuracy and efficiency of two approaches, GMYC and ABGD, in delimitating the three lineages. The result shows that ABGD is a better approach than GMYC. Our molecular data recognizes the three species as different populations of a single species, ranging from Taiwan Island to the continent. Therefore, we propose two new junior synonyms for *C. scutellaris*: *C. tamdaoensis*, **syn. nov.** and *C. elsae***syn. nov.**

## Introduction

The genus *Cyclommatus* Parry, 1863 (Lucanidae), includes some of the most striking stag beetles with enormous male mandibles and metallic body colorations. Many species display strong sexual dimorphism and male polymorphism, causing substantial confusion in morphological-based species delimitation. Phylogenetic analysis using molecular markers such as mitochondrial and nuclear genes can clarify many morphology-based species/subspecies’ taxonomic positions. Yet, in *Cyclommatus*, only a few such studies have been performed for a limited number of species (Kim and Farrell 2015; Tsai and Yeh 2016) and only mitogenomic data of *Cyclommatus vitalisi* Pouillaude, 1913 from southwestern China have been reported (Liu et al. 2017). To our knowledge, no multilocus phylogenetic analysis has been applied to resolve taxonomic debates in this genus to date.

**Figure 1.**
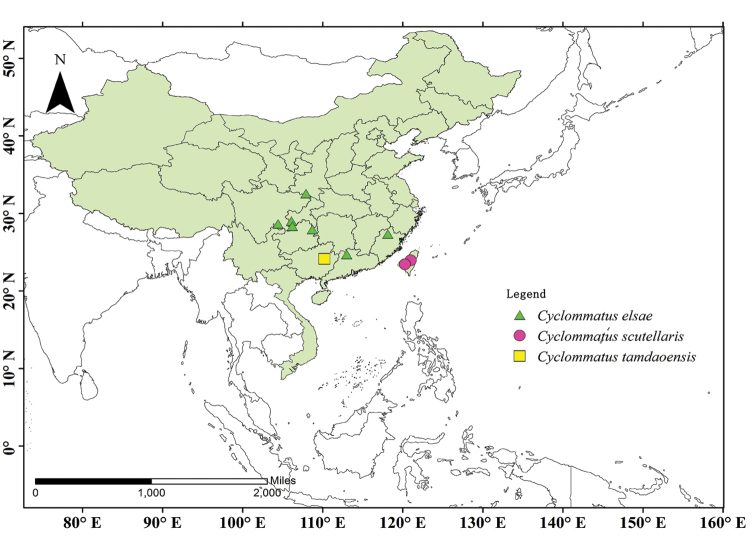
Collection localities of *C. scutellaris*, *C. elsae* and *C. tamdaoensis*. The map is generated using ARCGIS 10.2 software based on the geospatial data from the National Geomatics Center of China.

This study aims to resolve the long-debated taxonomic relationships among three *Cyclommatus* species, namely *C. scutellaris* Möllenkamp, 1912; *C. elsae* Kriesche, 1921 and *C. tamdaoensis* Fujita, 2010. *C. scutellaris* was first described by Möllenkamp (1912) and believed to be endemic to Taiwan Island. *Cyclommatus elsae* and *C. tamdaoensis* are distributed in southeastern China and the China-Vietnam border, respectively (Fig. 1). The three lineages’ taxonomic relationships were frequently revised by different studies, resulting in inconsistent conclusions (Möllenkamp 1912; Kurosawa 1974; Bomans 1979; Lacroix 1988; Maes 1992; Fujita 2010; Schenk and Nguyen 2015). Most of these revisions, however, lack sufficient data support. For instance, Fujita (2010) treated *C. scutellaris* and *C. elsae* as valid species and described the new taxon *C. tamdaoensis* from northern Vietnam. More recently, Huang and Chen (2017) considered them three subspecies based on morphological characters and considered *C. princeps* Schenk & Nguyen, 2015 from northern Vietnam as a junior synonym of *C. tamdaoensis*. Both studies used some key morphological diagnostic traits showing a considerable degree of intraspecific variabilities, such as the stripes on the pronotum and the male genitalia. The black lateral stripes in male individuals of *C. elsae* display two forms: the complete-form extending to the anterior angle of the pronotum and the partly patched form along the margins [see pl. 194, figs 8–2–2, 8–2–4 in Huang and Chen (2017)]. The black median stripe shows three forms: ranging from the broadly diamond shape to the reduced midline, then disappearing completely [see pl. 194, figs 8–2–2, 8–2–4 in Huang and Chen (2017)]. The lateral stripes of male *C. scutellaris* are patched and the middle one is absent, while those of *C. tamdaoensis* are the complete-form and diamond-shaped, respectively [see pl. 194, figs 8–1, 8–3 in Huang and Chen (2017)]. Moreover, characters of male genitalia, including the median lobes and the basal piece, which are very important in lucanid taxonomy (Holloway 2007), show variability within the samples of *C. elsae*, *C. scutellaris* and *C. tamdaoensis* [see pl. 196 in Huang and Chen (2017)]. The protibiae of males of all three taxa consistently show yellow setae along 3/5 of their inner margin [see pl. 194, figs 8–1–1, 8–2–1, 8–3–1 in Huang and Chen (2017)]. Finally, the females of all three taxa look uniform with broad lateral stripes and a diamond-shaped median stripe [see pl. 195 figs 8–1–4, 8–2–19, 8–3–4 in Huang and Chen (2017)].

The broadly distributed *C. elsae* displays substantial morphological variability within its range. Populations from Mt. Nanling in Guangdong are morphologically almost identical to *C. scutellaris*, whereas the Mt. Simianhshan (Chongqing) population are nearly indistinguishable from *C. tamdaoensis*. The three groups' intraspecific variations and interlineage morphological convergence suggest that they are likely different populations of the same species. Yet, the lack of molecular data makes it difficult to clarify their conspecific status. In the current study, we applied the multilocus approach to recognize the three species' relationship. Our goal is to resolve this long-debated issue in *Cyclommatus* species phylogeny while testing the multilocus molecular approach’s power and efficiency in resolving species delimitation problems for lucanid beetles.

## Materials and methods

### Taxon sampling

Thirty-three stag beetle specimens were examined, including 29 samples of *Cyclommatus* (15 *C. elsae*; 9 *C. scutellaris*; 5 *C. tamdaoensis*) as in groups, and 4 samples as outgroups (*C. albersi* Kraatz, 1894, *C. mniszechi* (Thomson, 1856); *C. nagaii* Fujita, 2010; *C. vitalisi*). Voucher specimens and their extracted genomic DNA are deposited in the research collection at the Museum of Anhui University, China (Suppl. material 1: Table S1).

### Laboratory protocols

The specimens were stored in 100% ethanol at -20 °C for molecular analysis. A small muscle was extracted from the sample using the Blood and Tissue Kit for total DNA extraction (Qiagen, Germany). Extracted DNA was stored at -20 °C until needed for the polymerase chain reaction (PCR). Four gene regions were targeted using the PCR. These regions included mitochondrial genes (16S rDNA, COI) and two nuclear genes (28S rDNA, Wingless). COI is often used in DNA barcoding because of its strong species identification ability (Han et al. 2016; Thormann et al. 2016). 16S rDNA is a conserved gene in animal mitochondria, which has a slow evolution rate. This gene is suitable for the study of distant taxa (Simon et al. 1994). COI and 16SrDNA are the most commonly used mitochondrial genes in the Lucanidae phylogeny (Tsai and Yeh 2016). 28S rDNA is relatively conservative in the evolutionary process and contains a highly variable region in the conserved sequence. It is also an excellent molecular marker to solve the phylogenetic relationship from species to families. Because wing pattern is a vital criterion for insect identification, Wingless is a necessary candidate gene for studying insect phylogeny. Still, the gene is only used in Lepidoptera and Coleoptera systematics (Liu and Jiang 2005). So, all specimens were sequenced for 16S rDNA, COI, 28S rDNA, and Wingless.

The primer sets used to amplify 16S rDNA, COI, 28S rDNA, and Wingless are shown in Table 1. PCR amplification reaction was carried out in a volume of 25 μL, in which 10 μM of each primer (forward and reverse) 1 μL, 2 μL template DNA, 12.5 μL 2×EasyTaq SuperMix (dye) and 8.5 μL sterile double distilled water (ddH2O) constituted a final volume of 25μL. PCR conditions were as followed: initial denaturation at 94 °C for 2 minutes, followed by 35–37 cycles of denaturation at 94 °C for the 40 s and annealing at 52–58 °C for 50s, and elongation at 70 °C for 50s, and then a final extension step at 72 °C for 7 min. The length of the fragments determined the annealing temperature. For sequencing, we used the ABI PRISM BigDye terminator version 3.1 sequencing kit (Life Technologies, USA) and cycle sequencing reactions were performed on ABI PRISM 3730xl automated sequencers (Life Technologies, USA) at Sangon Biotech Company, China. The sequences were submitted to GenBank with the accession number (Suppl. material 1: Table S1).

**Table 1.** List of the primer pairs and their respective reference used during the present study.

Gene	Primer name	Sequence (5'–3')	Reference
COI	COI-F1	CAACATTTATTTTGATTTTTTGG	Simon et al. (1994)
	COI-R1	TCCAATGCACTAATCTGCCATATTA	Simon et al. (1994)
16S rDNA	16S-F1	CCGGTTTGAACTCAGATCATG	Hosoya et al. (2001)
	16S-R1	TAATTTATTGTACCTTGTGTATCAG	Hosoya et al. (2001)
28S rDNA	28SDD	GGGACCCGTCTTGAAACAC	Monaghan et al. (2007)
	28SFF	TTACACACTCCTTAGCGGAT	Monaghan et al. (2007)
Wingless	Wg550F	ATGCGTCAGGARTGYAARTGYCAYGGYATGTC	Wild and Maddison (2008)
	WgAbRZ	CACTTNACYTCRCARCACCARTG	Wild and Maddison (2008)
	Wg578F	TGCACNGTGAARACYTGCTGGATG	Ward and Downie (2005)
	WgAbR	ACYTCGCAGCACCARTGGAA	Abouheif and Wray (2002)

### Phylogenetic analyses

Sequences were assembled in GENEIOUS PRIME 2019.1.1. All sequences were aligned in MEGA 7 (Kumar et al. 2016). Divergences among taxa were analyzed using MEGA 7.0 based on the Kimura 2-parameter model. DNA COI sequences of *Cyclommatus* were assembled for genetic distance analyses. Bayesian inference (BI) and Maximum Likelihood (ML) analyses were conducted using MRBAYES 3.2.7a and IQ-TREE web server, respectively. The BI and ML analysis was conducted on the CIPRES Science Gateway (Miller et al. 2011). Two independent chains starting from a random tree were run for 20,000 generations, sampling the tree every 10 generations. The initial 25% trees of each Markov Chain Monte Carlo (MCMC) chain were discarded as burn-in. A consensus tree was computed from the remaining 1500 trees combined from two runs, and the two runs converged at a maxdiff of less than 0.1. For ML analyses, the “automatic” option was set under the optimal evolution model, and the phylogenetic trees were constructed using an ultrafast bootstrap approximation approach with 10,000 replicates. Phylogenetic trees were viewed and edited in FIGTREE 1.4.4.

### Divergence time analysis

Divergence time was estimated with a relaxed clock Exponential model (Drummond and Rambaut 2007) in BEAST 2.6.0 (Bouckaert et al. 2014). The MCMC chain was run for 8.5×10^8^ generations, with four independent runs. Substitution rates of 1.77% per lineage in a million years (Myr) for COI combining with 0.54%/lineage/Myr for 16S rDNA have been suggested optimally for beetles (Papadopoulou et al. 2010). The resultant BEAST log files were viewed using TRACER 1.7 (Rambaut et al. 2018) to analyze the output results of the Effective Sample Size (ESS) for the posterior distribution of estimated parameter values. With a 25% burn-in threshold, all post-burn-in trees from the four independent runs were combined using the software LOG COMBINER 2.6.0 (Bouckaert et al. 2014). TREE ANNOTATOR 2.5.1 (Bouckaert et al. 2014) was used to summarize information (i.e., Nodal posterior probabilities, posterior estimates and highest posterior density limits) from the individual post-burn-in trees onto a single maximum clade credibility (MCC) tree. The summarized information was visualized on the MCC tree using FIGTREE 1.4.4.

### Species delimitation

We performed species delimitation using Automatic Barcode Gap Discovery (ABGD) and Generalized Mixed Yule Coalescent (GMYC) methods. The ABGD detects a gap in divergence distribution, which corresponds to differences between intraspecific and interspecific distances. When a gap exists, the process works well for species delimitation (Puillandre et al. 2012a). The ABGD analyses were performed at the webserver (https://bioinfo.mnhn.fr/abi/public/abgd/abgdweb.html). The following setting was used: steps (20), distance Kimura (K80) TS/TV (2.0), other parameter values employed defaults. GMYC delimits distinct genetic clusters by optimizing the set of nodes defining the transitions between inter-and intraspecific processes (Pons et al. 2006). The analysis was conducted using BEAST 2.6.0 under a relaxed clock Exponential model. ESS values assessed convergence. A burn-in with 25% was set to obtain an optimal consensus tree. The resulting tree was then used to analyze the data under the GMYC species delimitation approach in the software R with the package ‘splits ‘ using the single-threshold method (Ezard et al. 2015).

## Results

### Phylogenetic relationships

**Figure 2.**
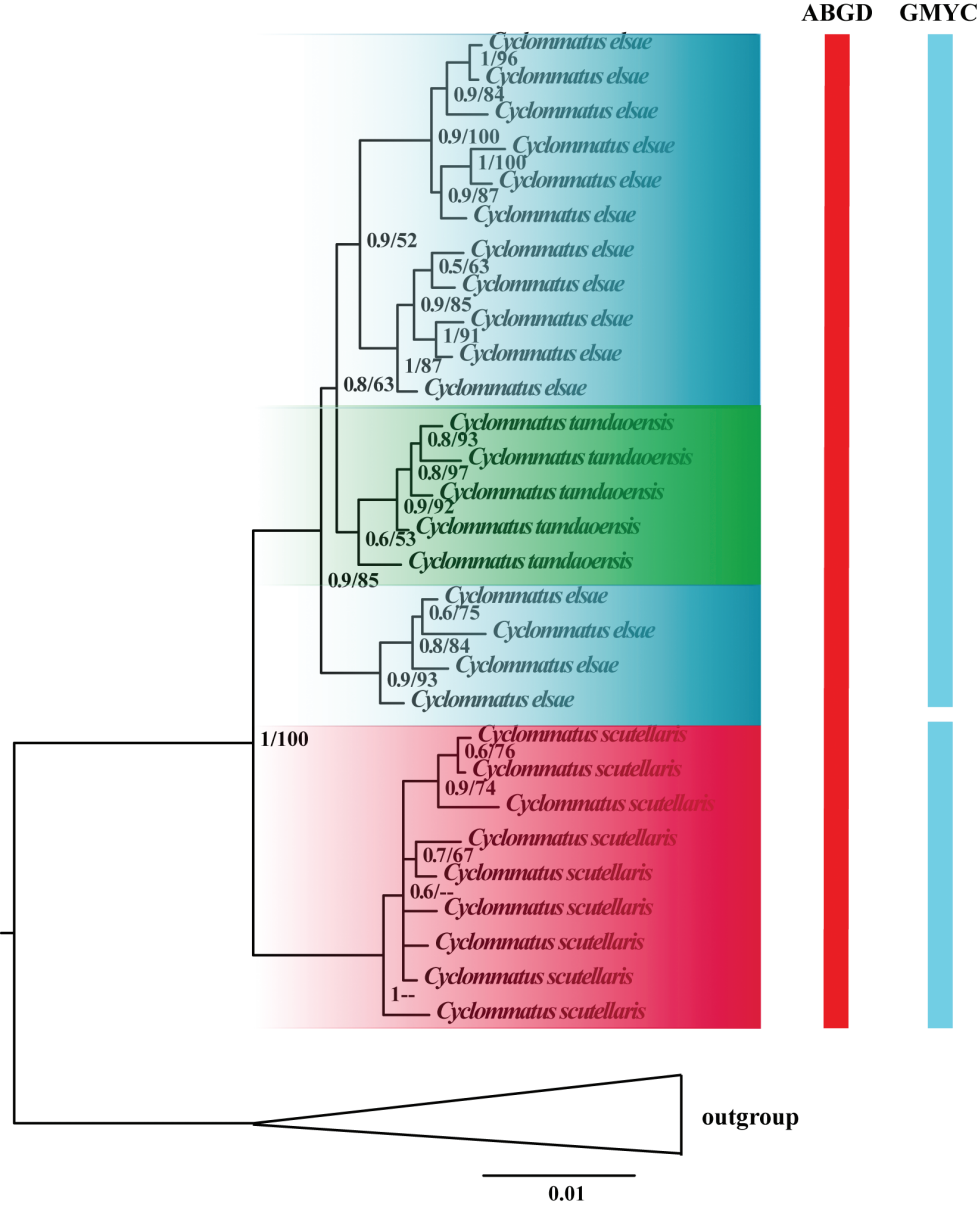
Bayesian topology showing the relationships within *C. scutellaris*, *C. elsae*, *C. tamdaoensis* and outgroups. Values next to each node represent Bayesian posterior probabilities (first number) and maximum likelihood bootstrap support (second number). The phylogenetic tree is based on Bayesian inference analysis of concatenated DNA sequence data from 16S rDNA, 28S rDNA, COI and Wingless. The columns on the right show numbers of entities identified by the ABGD, GMYC.

The BI and ML analysis showed consistent topology with a highly supported backbone (Fig. 2; Suppl. material 2: Fig. S1). The clade *C. scutellaris* was sister to *C. elsae* + *C. tamdaoensis* (Bayesian posterior probability, BPP = 1, Maximum Likelihood Bootstrap, MLB = 100). And the subclade *C. tamdaoensis* embedded in *C. elsae* (BPP = 0.7, MLB = 76). *Cyclommatus scutellaris* was an early branch in the three taxa and the species was monophyletic.

### Genetic distances

The genetic distances using the COI gene were calculated among the three taxa. The mean genetic distances between the *C. scutellaris*, *C. elsae* and *C. tamdaoensis* were no more than 0.035 (Table 2). The numbers were well below the minimum mean genetic distances of 0.122 among interspecific taxa of *Cyclommatus*.

**Table 2.** The mean genetic distances among all the taxa (Kimura 2-parameter)

	1	2	3	4	5	6
*C. albersi*						
*C. elsae*	0.159					
*C. mniszechi*	0.166	0.164				
*C. nagaii*	0.142	0.124	0.175			
*C. scutellaris*	0.166	0.035	0.169	0.129		
*C. tamdaoensis*	0.155	0.016	0.158	0.122	0.031	
*C. vitalisi*	0.152	0.150	0.145	0.146	0.159	0.146

### Divergence time analysis

**Figure 3.**
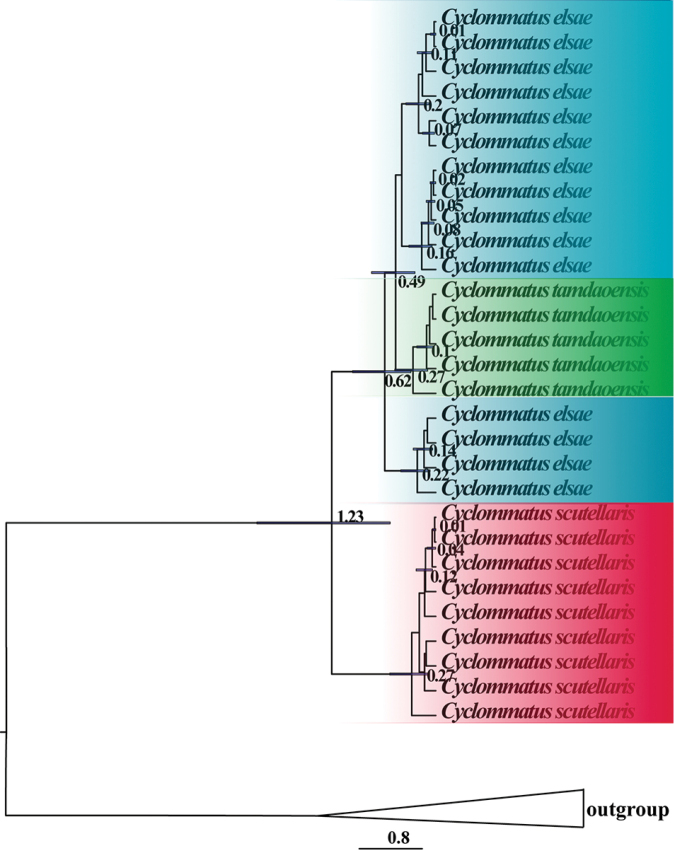
Maximum clade credibility time-tree obtained from BEAST based on COI and 16S rDNA. Divergence time estimates are represented next to the nodes (in millions of years) with horizontal bars indicating 95% highest posterior density intervals.

Based on the COI gene and 16S rDNA gene, calibration time was analyzed to describe these taxonomically controversial species’ possible differentiation history. The analysis under BI converged well as all parameters had ESS values above 200. The mean divergence age estimates and 95% High Posterior Density (HPD) for nodes of interest-based on the BEAST analysis are presented in Fig. 3, and Suppl. material 3: Fig. S2. Among the three taxa, *C. scutellaris* diverged during the Middle Pleistocene circa 1.23 million years ago (Mya) (95% HPD: 0.58–2.28 Mya). Within the clade *C. elsae* + *C. tamdaoensis*, the crown node’s age estimate was dated to be 0.62Mya (95% HPD: 0.32–1.06 Mya) in the Late Pleistocene. The age of *C. tamdaoensis* was estimated to be 0.49 Mya (95% HPD: 0.27–0.82 Mya) in the Late Pleistocene.

### Species delimitation

Two species delimitation methods were employed to evaluate which one was most suitable for multilocus phylogenetic analyses. ABGD analyses suggested the three taxa were one molecular operational taxonomic unit (MOTU), whereas GMYC divided *C. scutellaris*, *C. elsae* and *C. tamdaoensis* into two MOTUs (Fig. 2).

### Taxonomic account

#### Cyclommatus scutellaris

Taxon classificationAnimaliaColeopteraLucanidae

Möllenkamp, 1912

D7A66451-4E43-5834-A862-F03EA0276718

Cyclommatus scutellaris Möllenkamp, 1912: 7.
Cyclommatus elsae Kriesche, 1921: 95. syn. nov.
Cyclommatus tamdaoensis Fujita, 2010:14. syn. nov.
Cyclommatus princeps Schenk & Nguyen, 2015: 7. (Synonymized as a junior synonym of Cyclommatus tamdaoensis by Huang and Chen 2017: 193)

##### Material examined.

China • 1 male; Yunnan Province, Shuifu County; 27 Jul. 2010; JS Xu and LX Chang leg. • 1 male; Shaanxi Province, Zhenba County; 10 Jul. 1996. • 3 males; Chongqing Province, Mt. Simianshan; 29 Aug. 2014. • 3 males; Guizhou Province, Mt. Fanjingshan; 29 Jul. 2014; YF Wu. • 1 male; Guizhou Province, Xishui County, 18 Jul. 2015, LX Zhu. • 1 male; Fujian Province, Mt. Wuyishan; 18 Jul. 2011; Q Zhang and YY Cao. • 1 male; same locality as for preceding; 18 Aug.2010; F Zhong and XY Hu. • 3 males; Taiwan Island; 29 Aug. 1995. • 1 male; same locality as for preceding; 17 May 2008. • 1 male; same locality as for preceding; 5 Oct. 2008. • 2 males; same locality as for preceding; 20 Jul. 2010; • 2 males; same locality as for preceding; 21 Jul. 2011. • 3 males; Guangdong Province, Mt. Nanling; 18 Aug. 2010; HY Liu. • 1 male; same locality as for preceding; 9 Aug. 2011, F Zhong; • 4 males; Guangxi Autonomous Region, Jinxiu County; 18 Jul. 2012. • 1 male; same locality as for preceding; 28 Jun. 2013.

##### Diagnosis.

The species is characterized in the male by the matt dorsal surface of the entire body and the long seta-range on the inner margin of the protibial, and in the female by the elytra usually without black stripes. The female can be distinguished from other members of *Cyclommatus* by the following combination of characters:1) ground color of the dorsal surface of the body redder; 2) canthus often short, with lateral end blunt, and usually with a convex or straight outer margin; 3) central black band on the pronotum clearly defined and often narrower than the lateral orange band (Huang and Chen 2017).

##### Distribution.

China (Yunnan, Shaanxi, Chongqing, Guizhou, Fujian, Taiwan Island, Guangdong, Guangxi).

## Discussion

This study presents consistent phylogenetic relationships inferred by the BI and ML methods. The trees’ identical topology strongly supports that *C. scutellaris* is sister to the clade *C. elsae* +*C. tamdaoensis*. The nested structure of *C. elsae* and *C. tamdaoensis* suggests that the two nominal taxa should be treated as one species. The genetic distance also indicates that *C. elsae* and *C. tamdaoensis* are most likely two continental populations of the Taiwanese species *C. scutellaris*.

The divergence time estimation shows that *C. scutellaris* began to diverge from the Middle Pleistocene with subsequent deep genetic isolation during the Late Pleistocene. During the glacial maxima, a land bridge facilitated the contraction of the geographical ranges of some species southwards into Taiwan (Kawamura et al. 2016). Once the glaciations ended, the rising sea levels resulted in vicariant isolation. The relict populations might have retreated to the montane habitat of Taiwan Island, causing the present disjunct distributions (Päckert et al. 2011; Wang et al. 2013). Many studies interpreted the global cooling and aridification, the Qinghai–Tibet Plateau uplift and the abrupt climate change in East Asia during the Neogene and Quaternary as responsible for the speciation, intraspecific divergence, genetic diversification, and significant phylogeographic break between the western and eastern clade in Chinese fauna (Meng et al. 2015; Qiu et al. 2017; Ye et al. 2017; Zhou et al. 2017).

Accurate species delimitations are critical in many biology areas, such as conservation biology (designating endangered species) and evolutionary biology (describing diversification patterns). Traditionally, species are identified and described by morphological characters. However, using morphological data alone may underestimate the number of species (Yang and Rannala 2010). Therefore, molecular data are used to delimitate species in the present study. However, GMYC and ABGD methods obtained different results. GMYC has been developed to delimit species according to single-locus data, which has a strong theoretical basis. Although the method is based on a reliable phylogenetic framework, it largely depends on the ultrametric gene tree’s correctness. Errors in the framework underpinning the analysis may affect the final results (Kekkonen and Hebert 2014). Also, GMYC usually over-splits species, mainly due to low genetic divergence between lineages and overlap of interspecific and intraspecific divergences, or due to lack of reciprocal monophyly between sister clades (Talavera et al. 2013; Pentinsaari et al. 2016; Stokkan et al. 2018). Evaluations of the accuracy and efficiency of ABGD conducted by different researchers reached a consensus that ABGD is more conservative and faster than other methods (Puillandre et al. 2012a; Puillandre et al. 2012b; Kekkonen and Hebert 2014; Guillemin et al. 2016). Our results also produced a consistent conclusion. ABGD produced one MOTU, which agrees with the number of species defined based on multilocus phylogenetic analyses.

In summary, our study resolved a long-standing debate on species recognition in the genus *Cyclommatus* while illustrating the necessity of employing multiple data types, both morphological and molecular, for efficient and accurate species delimitation in taxa with a high degree of phenotypic convergence and variations.

## Supplementary Material

XML Treatment for Cyclommatus scutellaris

## Supplementary materials

Supplementary material 1 Table S1. A list of specimens’ voucher information and GenBank accession numbers used in this study Data type phylogenetic https://binary.pensoft.net/file/515388This dataset is made available under the Open Database License (http://opendatacommons.org/licenses/odbl/1.0/). The Open Database License (ODbL) is a license agreement intended to allow users to freely share, modify, and use this Dataset while maintaining this same freedom for others, provided that the original source and author(s) are credited.Jiao Jiao Yuan

Supplementary material 2 Figure S1. Bayesian topology showing the relationships within *C. scutellaris*, *C. elsae*, *C. tamdaoensis* and outgroups Data type phylogenetic Explanation note The figure also indicates outgroups and accession numbers. The columns on the right show numbers of entities identified by the ABGD, GMYC. https://binary.pensoft.net/file/515389This dataset is made available under the Open Database License (http://opendatacommons.org/licenses/odbl/1.0/). The Open Database License (ODbL) is a license agreement intended to allow users to freely share, modify, and use this Dataset while maintaining this same freedom for others, provided that the original source and author(s) are credited.Jiao Jiao Yuan

Supplementary material 3 Figure S2. Maximum clade credibility time-tree based on COI and 16S rDNA Data type phylogenetic Explanation note Age estimates are indicated at the nodes (Mya). The outgroups are shown in the figure. https://binary.pensoft.net/file/515390This dataset is made available under the Open Database License (http://opendatacommons.org/licenses/odbl/1.0/). The Open Database License (ODbL) is a license agreement intended to allow users to freely share, modify, and use this Dataset while maintaining this same freedom for others, provided that the original source and author(s) are credited.Jiao Jiao Yuan
