# Supplementary figures and images for: A multilocus assessment reveals two new synonymies for East Asian Cyclommatus stag beetles (Coleoptera, Lucanidae)
Source: Zookeys. 2021 Mar 2;1021:65–79. doi: 10.3897/zookeys.1021.58832 (PMC7943532; doi:10.3897/zookeys.1021.58832)

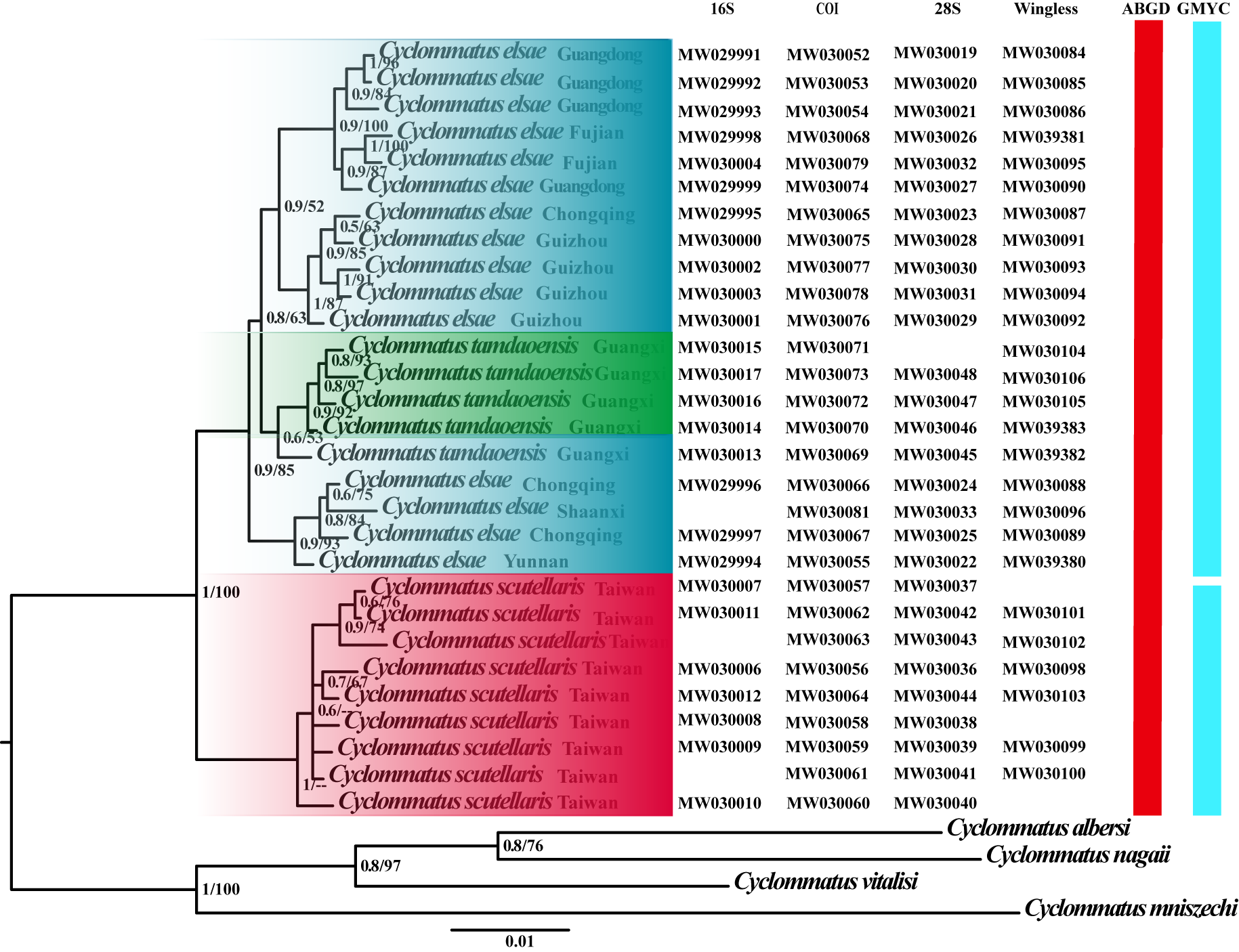

Supplement: Supplementary material 2 — Figure S1. Bayesian topology showing the relationships within C. scutellaris, C. elsae, C. tamdaoensis and outgroups [file zookeys-1021-065-s002.tif]

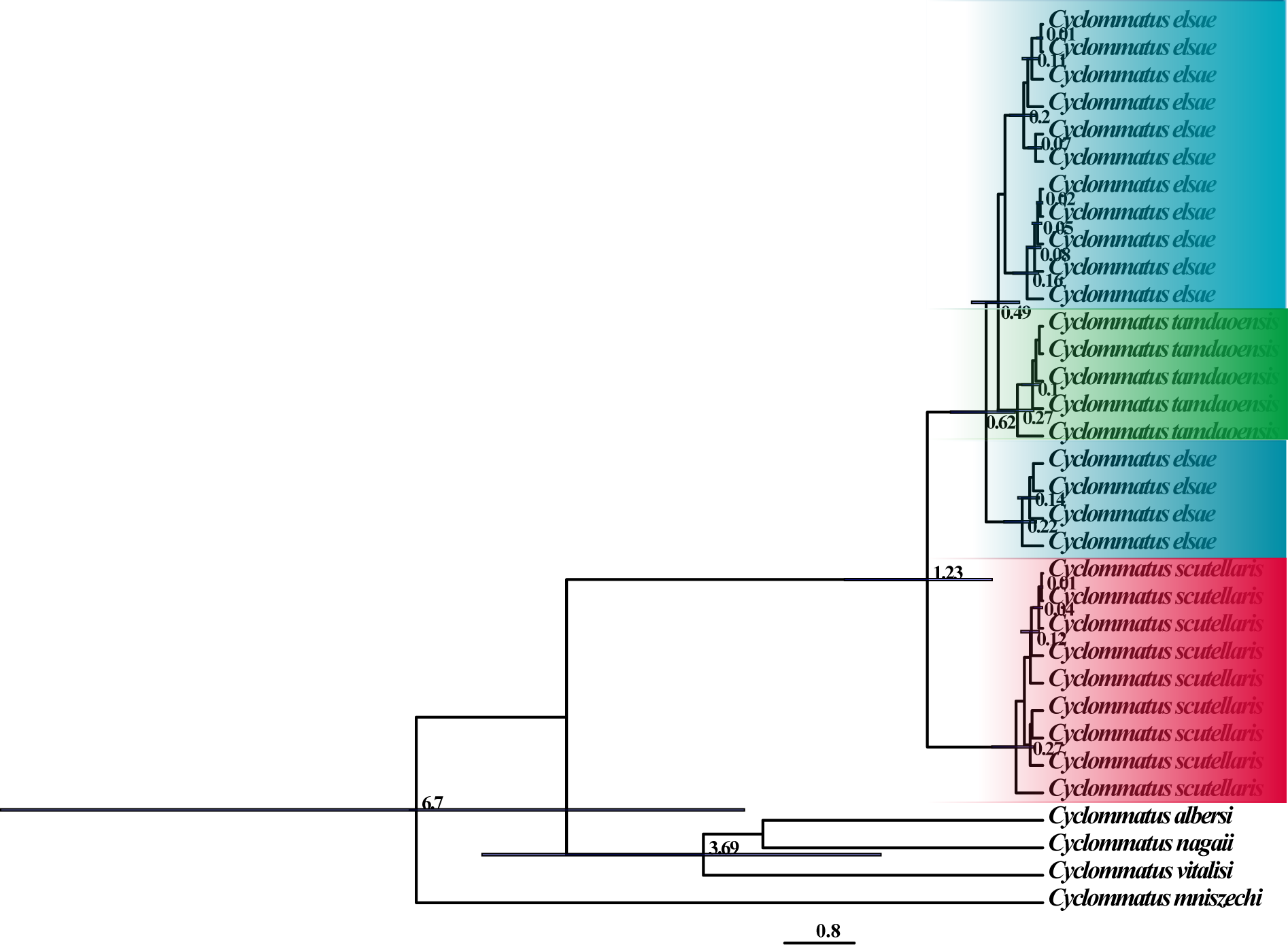

Supplement: Supplementary material 3 — Figure S2. Maximum clade credibility time-tree based on COI and 16S rDNA [file zookeys-1021-065-s003.tif]
